# A retrospective study: Can dual ART mitigate the risk of potential drug-drug interactions among PLWH under stable ART?

**DOI:** 10.55730/1300-0144.5718

**Published:** 2023-09-17

**Authors:** Ahmet Çağkan İNKAYA, Fatma Nisa BALLI, Emre KARA, Kutay DEMİRKAN, Serhat ÜNAL

**Affiliations:** 1 Department of Infectious Diseases and Clinical Microbiology, Faculty of Medicine, Hacettepe University, Ankara, Turkiye; 2 Department of Clinical Pharmacy, Faculty of Pharmacy, Gazi University, Ankara, Turkiye; 3 Department of Clinical Pharmacy, Faculty of Pharmacy, Hacettepe University, Ankara, Turkiye

**Keywords:** Antiretroviral, drug-drug interaction, dual therapy, HIV, polypharmacy

## Abstract

**Background/aim:**

People living with human immunodeficiency virus (PLWH) are getting older. Age-related comorbidities in PLWH result in polypharmacy and increase the risk for potential drug-drug interactions (pDDIs). This study aimed to evaluate how the rate of pDDIs would change if the treatment of patients receiving different combined antiretroviral therapies (ARTs) were theoretically changed with dolutegravir/lamivudine (DTG+3TC) or cabotegravir/rilpivirine (CAB+RPV).

**Materials and methods:**

This study was conducted at the infectious disease outpatient clinic of a university hospital as a follow-up of a previous study. The data of PLWH receiving at least 1 comedication other than antiretrovirals (ARVs) were retrospectively reviewed and analyzed. The Drugs.com/Drug Interactions Checker and University of Liverpool HIV Drug Interactions Checker databases were used to identify pDDIs and their severities.

**Results:**

A total of 75 PLWH, of whom 83% were male, with a mean age (± standard deviation) of 46.5 (±12.98) years were included. Polypharmacy was observed in 59 (79%) of the participants; however, with dual ARV options, the probability of polypharmacy was 35 (47%) (p < 0.001). In the Drugs.com database, no significant difference was found in terms of pDDIs between the treatment of current ARTs (64%) and DTG/3TC (%44) (p = 0.06) or CAB/RPV (%64) (p = 0.521). However, in the University of Liverpool database, the current rate of pDDIs (55%) was significantly higher compared to the theoretical treatment of DTG/3TC (40%) (p = 0.029), oral CAB/RPV (48%) (p = 0.003), and injectable CAB/RPV (31%) use (p = 0.006).

**Conclusion:**

The results suggest that dual treatment regimens can reduce pDDIs, resulting in better tolerance and probably higher quality of life among PLWH.

## 1. Introduction

Since the introduction of antiretroviral therapy (ART), the survival of people living with human immunodeficiency virus (PLWH) has become close to that of uninfected people [1]. For 2 decades, triple antiretroviral (ARV) drug combinations constituted the mainstay of HIV treatment. Achievement in drug development has resulted in more potent and tolerable ARVs, single-tablet regimens, and long-acting formulas [2].

Treatment costs, adverse events, and drug adherence pose major challenges in the care of PLWH [2, 3]. Aging with HIV is a growing concern, because as the people age, the number of comorbidities as well as comedications increases sharply [4, 5]. Furthermore, ARV combinations, including more medications, are prone to more drug-drug interactions (DDIs) when compared to regimens with lower chemical moieties. Thus, many pharmaceutical companies are investing in medications with a lower number of formulas and long-acting agents. Dual treatment regimens would save costs, reduce long-term drug toxicities, facilitate drug adherence, and have less propensity for DDIs [6, 7].

A 2-drug combination of dolutegravir + lamivudine (DTG+3TC) or cabotegravir + rilpivirine (CAB+RPV) seems promising as both regimens effectively suppress the viral load while preserved immunological recovery besides is a safe and convenient alternative [6].

This study aimed to evaluate how the rate of potential DDIs (pDDIs) would change if the ongoing ART of PLWH were theoretically switched to DTG+3TC or CAB+RPV.

## 2. Materials and methods

### 2.1. Study design and population

This retrospective observational study was carried out at the Infectious Diseases Outpatient Clinic of Hacettepe University Adult Hospital between September 1st, 2015, and July 1st, 2016. The characteristics of the study population and setting were previously described by Kara et al. [8]. Inclusion criteria were being 18 years of age or older, receiving ART for at least 3 months, and not being involved in any clinical trial during the study period. Exclusion criteria were not being on any comedication at the time of the survey, and having hepatitis B coinfection (as documented by a positive HBsAg with ELISA) or having previously been exposed to HBV (as documented by positive anti-HBc IgG with ELISA). The data were reanalyzed to explore whether potential DDIs would change after switching to dual ARV regimens including, 3TC/DTG or CAB/RPV.

### 2.2. Data collection

Online patient management software (Nucleus) and printed patient files were used to collect data. The ARTs were analyzed by replacing them with dual treatment options (DTG+3TC or CAB+RPV). Comedications were evaluated in the same way without changing them. Any pDDIs were identified using the online Drugs.com/ Interaction Checker (https://www.drugs.com/drug_interactions.html) and Liverpool HIV Drug Interaction Checker (www.hiv-druginteractions.org) databases. Parenteral use of CAB+RPV has been considered a separate option in the University of Liverpool database, but parenteral use could not be evaluated separately on the Drugs.com database. The Drugs.com database was developed to evaluate interactions between drugs without being specific to any disease. The University of Liverpool database was only used to evaluate interactions between ARV drugs and other drugs. In this study, only the interactions of ARV drugs with comedications were evaluated, but not the interactions within the comedications. Lercanidipine, gliclazide, domperidone, and 4-aminosalicylic acid are not included in the Drugs.com database; zinc and ferrous sulfate are not included in the University of Liverpool database; and barnidipine, zofenopril, dexketoprofen, and ornidazole are not included in either database. Thus, these drugs were not evaluated in terms of pDDIs.

Herein, polypharmacy was defined as the routine use of 5 or more different comedications^1^. The study protocol was evaluated and approved by the Hacettepe University Non-Interventional Ethics Committee (decision number: GO-15/558-14).

### 2.3. Statistical analysis

The data were evaluated using descriptive statistics after normality analysis. The categorical data analysis of 2 independent groups was performed using the chi squared or Fisher exact tests. The Mann-Whitney U test was used for the nonparametric variables. The Kruskal-Wallis test was used when the assumption could not be obtained for more than 2 groups. Statistical significance was accepted as p = 0.05. The statistical analysis was performed using IBM SPSS Statistics for Windows 23.0 (IBM Corp., Armonk, NY, USA).

## 3. Results

Of the 181 PLWH, 75 were on at least 1 comedication and included in the final analysis. Demographics of the study group are given in Table 1. Polypharmacy was observed in 59 (79%) participants. However, with dual ARV options, the probability of polypharmacy was observed in 35 (47%) participants (p < 0.001).

According to the Drugs.com database, no significant difference was found in terms of pDDIs between continuing the current ART and switching to DTG/3TC (p = 0.06) or CAB/RPV (p = 0.521) (Table 2). When the pDDIs were explored with the University of Liverpool database, continuing the current ART regime was related to significantly higher pDDIs when compared to the theoretical switch to DTG/3TC (p = 0.029), oral CAB/RPV (p = 0.003), and injectable CAB/RPV (p = 0.006) (Table 2). The rate of pDDIs with oral CAB/RPV (48%) was significantly higher than that with injectable CAB/RPV (31%) (p < 0.001) according to the University of Liverpool database.

A total of 107 pDDIs (29 major, 64 moderate, 14 minor) were reported by the Drugs.com database for those who continued their existing ART regime. In the theoretical switch to DTG/3TC and oral CBV/RPV scenario, 38 pDDIs (11 major, 11 moderate, 16 minor) and 82 pDDIs (29 major, 43 moderate, 10 minor) were identified, respectively.

A total of 81 pDDIs (4 did not coadminister, 46 potential interactions, 31 potential weak interactions) were reported by the University of Liverpool database for those who continued their existing ART regime. In the theoretical switch to DTG/3TC, oral CBV/RPV and injectable CAB/RPV scenario, 34 pDDIs (23 potential interactions, 11 potential weak interactions), 47 pDDIs (11 did not coadminister, 28 potential interactions, 8 potential weak interactions), and 28 pDDIs (2 did not coadminister, 18 potential interactions, 8 potential weak interactions) were identified, respectively.

Ritonavir (28%) and tenofovir disoproxil fumarate (TDF) (16%)-based regimens were found to have the highest potential for pDDIs according to the Drugs.com database. Moreover, the University of Liverpool database identified ritonavir (27%), TDF (13.5%), darunavir/r (13.5%), and cobicistat (13.5%) as possessing the highest potential for pDDIs. According to the pDDI outputs, the coadministration of TDF may potentiate nephrotoxicity. Details of the most common (≥10% in at least 1 interaction database) pDDI pairs in the databases are presented in Table 3.

## 4. Discussion

The paradigm of 3-drug ART is changing day by day as the 2-drug treatment regimens are proved to be effective, safe, and enduring options. The results obtained herein strengthen the notion that the lower the number of medications, the lower the chance of pDDIs. There are inconsistencies regarding DDI definitions between different databases. Results of the current analysis indicated that the risk of drug interactions is reduced by 27% (55% to 40%) with DTG/3TC, 13% (55% to 48%) with oral CAB, and 44% (55% to 31%) with injectable CAB according to the University of Liverpool database.

A very recent analysis from Italy showed that switching to DTG/3TC lowered pDDIs from 92 to 12 in the elderly population (aged >65) [9]. The results of this current study are in line with the results of Calza et al. The concept of ‘deprescribing’ is gaining ground in the management of multimorbid-multicomplex patients. Deprescribing a third ARV drug is associated with lower pDDIs. Moreover, managing comorbidities with a low chemical burden, not adding a drug to manage possible drug-related problems, and discontinuing a drug, which in the future, may lead to cholinergic suppression and possible anticholinergic effects are also classified under the term of deprescribing [10–12].

The bioavailability of DTG, like other oral integrase (IN) strand transfer inhibitor (INSTI) agents, can be affected by metal cation coadministration. The peak DTG plasma concentration and area under the receiver operating characteristic curve can be decreased up to 40% and 60% when coadministered with calcium carbonate and ferrous fumarate, respectively [13]. Moreover, the awareness of potential drug interactions between oral metal cations and DTG by physicians was reported to be limited in a high endemic region, which might hinder treatment success [14]. The results obtained in the current study indicated that DDIs associated with DTG/3TC are limited to food intake (related to calcium) and metformin treatment, both of which can easily be handled with dietary and dosage adjustments. Administering an ART regimen through injection may lower the chance of treatment failures because of the chelation effect related to oral cation intake. CAB/RPV has been formulated in oral and injectable formulations. The oral formulation of CBV is prone to food interactions that can be overcome by injectable strategies. As a precaution for prolonged allergic reactions with long-acting formulations, CAB/RPV was initially administered orally and switched to injectable formulations after the first month of treatment [15]. However, both the oral formulation as well as the long-acting injectable formulation have been tolerated well and proven to be safe in randomized controlled trials and real-life experiences [16, 17]. In the present study, oral CBV/RPV interacted with oral calcium supplementation, and this interaction disappeared in the injectable regimes. Given that injectable CBV/RPV is well tolerated and safe, skipping the oral lead-in phase can be considered as a first line strategy to lower the risk of food-drug interactions [15]. Moreover, it was demonstrated that skipping the oral lead-in phase was associated with the experience of physicians in clinical trials and the origin of their country of practice (Europe vs. North America). Favorable effects on pDDIs may also affect the attitudes of physicians about omitting the oral lead-in phase [18]. A lower chance of pDDIs and a lack of allergic reactions to CAB/RPV may encourage physicians to start with the injection strategy.

The last 4 decades of the struggle with HIV has mainly focused on viral suppression and immunological recovery. With the advent of potent new ARV drugs, those goals have already been met by numerous ARV regimes [6]. All that remains is the fourth 95 of the Joint United Nations Programme on HIV/AIDS’ ambitious treatment target: the quality of life (QoL) for PLWH. Improvement in QoL can only be achieved with a holistic approach, including but not limited to effective comorbidity management and deprescribing [19, 20]. Yoshino et al. showed that switching to the DTG/3TC regimen was associated with improvements in the Pittsburgh Sleep Quality Index global score and mental component summary scores [21]. Contradictorily, a recent study by Figueroa et al. explored whether dual drug regimens are associated with favorable QoL in PLWH. In their randomized controlled trial, improvement in QoL was compared between dual and standard triple-agent regimens. At the end of the 48-week follow up, mental- and physical health-related QoL recovered in both groups [22]. However, in their study, the dual treatment was composed of darunavir/ritonavir + lamivudine, which is definitively not a 2-drug regimen. Furthermore, ritonavir included in the ART regime has notoriously been associated with extensive pDDIs [23, 24]. Moreover, protease sparing regimens have been associated with improved QoL in PLWH [25]. Relying on the aforementioned data, it can be argued that dual treatment with INSTIs drugs will affect the reported outcomes of patients and ease their compliance to the treatment.

DTG/3TC is a potent dual drug regimen shown to be effective and safe for naïve treatment as well as for experienced patients and has been endorsed as a first line regime by American, European, and Turkish guidelines^2^,^3^,^4^. Because of concerns about antiviral resistance, DTG/3TC was initially licensed in the treatment of patients without transmitted antiviral resistance^5^. However, recent reports from different settings have shown promising evidence for its effectiveness in the treatment of people with the M184V mutation [26, 27]. Switching to DTG/3TC in suppressed patients, regardless of a prior resistance profile, will ensure viral suppression while limiting the DDI risks and therefore, improve QoL for PLWH. However, a recent report from the LAMRES study group demonstrated concerns over DTG/3TC efficacy in people with preexisting M184V mutations. In their study, virological failure was pronounced in those with a shorter duration of viral suppression, whereas PLWH with suppressed viremia longer than 3.5 years rarely experienced virological failures. On the other hand, they could not demonstrate the effect of viral suppression in the multivariable model [26]. Regardless, before further evidence becomes available, all attending physicians should be vigilant about the duration of viral suppression and patient compliance on the ART.

This is the first study from Türkiye exploring whether dual treatment options will be related to better DDI profiles. The cohort herein was composed of heavily treated and multimorbid PLWH under polypharmacy. The results showed that deprescribing the fourth or even the third ARV will lower the prevalence of DDIs among PLWH. This study was limited by its retrospective, hypothetical single-center design. Moreover, regimes containing all triple ARVs were not covered and the dual-treatment effect on QoL was not demonstrated.

Achieving the ultimate goal of QoL among PLWH necessitates a holistic approach to comorbidities. Given that DTG/3TC is effective even in the presence of underlying 3TC resistance and associated with favorable efficacy, lowering the number of ARVs (presumably) will not speed up the process sof viral suppression. The results herein showed that dual treatment regimens are associated with better DDI profiles, which will culminate in better tolerance and probably higher QoL among PLWH.

## Figures and Tables

**Table 1:** Demographic characteristics of the PLWH.

Age, mean ± standard deviation	46.5 (±12.98)
≥50	34 (45)
<50	41 (55)
Sex, n (%)	
Male	62 (83)
Female	13 (17)
PLWH with ≥1 comorbidity, n (%)	51 (68)
Number of comorbidities, median (min–max)	1 (0–5)
Most commonly reported (≥10%) comorbidities, n (%)	
Hypertension	23 (31)
Dyslipidemia	15 (20)
Depression	13 (17)
Diabetes mellitus	12 (16)
Number of medications, median (min–max)	6 (4–11)
Most commonly reported (≥10%) medications by patients, n (%)	
TDF	70 (93)
FTC	70 (93)
DTG	18 (24)
EVG/r	15 (20)
DRV	14 (19)
RAL	14 (19)
EFV	13 (17)
Number of comedications, median (min–max)	2 (1–7)
Most commonly reported (≥10%) comedications by patients, n (%)	
Cholecalciferol	12 (16)
Metformin	11 (15)
Cotrimoxazole	10 (13)
Rosuvastatin	10 (13)
Acetylsalicylic acid	9 (12)
Azithromycin	9 (12)
Escitalopram	8 (11)
Calcium carbonate	8 (11)

FTC, emtricitabine; TDF, tenofovir disoproxil fumarate; r, ritonavir; DTG, dolutegravir; EVG, elvitegravir; DRV, darunavir; RAL, raltegravir; EFV, efavirenz.

**Table 2:** Comparison of current therapy and dual treatment options in terms of pDDIs.

Databases	Incidence of pDDIs, n (%)						
	Current ARTs	DTG/3TC	p-value	CAB/RPV oral	p-value	CAB/RPV injectable	p-value
Drugs.com	48 (64%)	33 (44%)	0.06	48 (64%)	0.521	-	-
University of Liverpool	41 (55%)	30 (40%)	0.029	36 (48%)	0.003	23 (31%)	0.006

pDDI, drug-drug interaction; DTG/3TC, dolutegravir/lamivudine; CAB/RPV, cabotegravir/rilpivirine.

**Table 3:** Details of the most commonly reported (≥10% in at least 1 drug interaction database) pDDI pairs in drug interaction databases.

ARV regimen	pDDI pairs		Databases			
	ARV	non-ARV	Drugs.com/Drug Interactions Checker	N (%)	University of Liverpool HIV Drug Interactions Checker	N (%)
DTG/3TC	DTG	Metformin	Moderate	11 (29)	Potential	11 (32)
	3TC	TMP	Minor	10 (26)	Potential weak	10 (29)
	DTG	Calcium carbonate	Major	8 (21)	Potential	8 (23.5)
	DTG	INH	Minor	5 (13)	No interaction expected	-
CAB/RPV oral	RPV	SMX	Minor	10 (12)	No interaction expected	-
	RPV	Azithromycin	Moderate	9 (11)	Potential	9 (19)
	CAB	Calcium carbonate	Major	8 (10)	Potential	8 (17)
	RPV	Escitalopram	Major	7 (8.5)	Potential	7 (15)
	RPV	Calcium carbonate	Moderate	8 (10)	No interaction expected	-
CAB/RPV injectable	RPV	Azithromycin	-	-	Potential	9 (32)
	RPV	Escitalopram	-	-	Potential	7 (25)

c, cobicistat; INH, isoniazid; SMX, sulfamethoxazole; TMP, trimethoprim

## Footnotes

1 World Health Organization (2019). Medication safety in polypharmacy: Technical report [online]. Website: https://www.who.int/publications/i/item/WHO-UHC-SDS-2019.11 [accessed 01 09 2023].

2 European AIDS Clinical Society (2022). EACS Guidelines Version 11.1 [online]. Website https://www.eacsociety.org/media/guidelines-11.1_final_09-10.pdf [accessed 01 09 2023].

3 Department of Health and Human Services. Panel on Antiretroviral Guidelines for Adults and Adolescents (2023). Guidelines for the Use of Antiretroviral Agents in Adults and Adolescents with HIV [online]. Website https://clinicalinfo.hiv.gov/sites/default/files/guidelines/documents/adult-adolescent-arv/guidelines-adult-adolescent-arv.pdf [accessed 01 09 2023].

4 Türk HIV/AIDS Platformu (2021). HIV/AIDS Tanı İzlem ve Tedavi El Kitabı Sürüm 2.0. (in Turkish) [online]. Website http://www.aidsvecinselhastaliklar.com/uploads/files/HIV%3AAIDS%20El%20Kitab%C4%B1%282%29.pdf [accessed 01 09 2023].

5 Research Triangle Park, NC: ViiV Healthcare (2019). Dovato (dolutegravir and lamivudine) (Package Insert) [online]. Website https://www.accessdata.fda.gov/drugsatfda_docs/label/2019/211994s000lbl.pdf [accessed 01 09 2023].
